# Characterizing the efficacy of zuranolone across patient subgroups and symptom domains in Japanese patients with major depressive disorder: a pooled analysis of two placebo-controlled trials

**DOI:** 10.3389/fpsyt.2026.1891881

**Published:** 2026-10-22

**Authors:** Masaki Kato, Takamichi Baba, Takuhiro Sonoyama, Nakao Iwata

**Affiliations:** 1 Department of Neuropsychiatry, Kansai Medical University, Osaka, Japan; 2 Drug Development and Regulatory Science Division, Shionogi & Co., Ltd., Osaka, Japan; 3 Department of Psychiatry, Fujita Health University School of Medicine, Aichi, Japan

**Keywords:** Japan, major depressive disorder, pooled analysis, subgroup, symptom domain, zuranolone

## Abstract

**Aim:**

Zuranolone has been reported to reduce the 17-item Hamilton Rating Scale for Depression (HAM-D17) total score as early as Day 3 in Japanese patients with major depressive disorder (MDD), with the reduction in the HAM-D17 total score being maintained after the treatment period. This *post hoc* pooled analysis aimed to evaluate the treatment effects of zuranolone across baseline characteristics and to characterize how changes in specific symptom domains—core, anxiety, and insomnia—were associated with the early reduction in the HAM-D17 total score and its subsequent trajectory, using pooled data from two randomized, double-blind, placebo-controlled Phase 2 and Phase 3 trials.

**Methods:**

Data from the Phase 2 and Phase 3 trials were combined for efficacy and safety analyses. Participants were randomly assigned to receive either oral zuranolone or placebo daily for 14 days and followed up for 6 weeks. The endpoints included the change from baseline in the HAM-D17 total score, response and remission rates, subgroup analyses, and subscale scores (core, anxiety, and insomnia) and their relative contributions to the between-group difference (zuranolone vs. placebo) in the HAM-D17 total score.

**Results:**

The pooled full-analysis set included 284 and 278 participants in the zuranolone and placebo groups, respectively. The treatment effects were consistent across subgroups defined by baseline severity, sex, episode recurrence, and other characteristics. The HAM-D17 subscale analyses indicated reductions in scores across the core, anxiety, and insomnia subscales during the treatment period, with reductions in the insomnia subscale score accounting for the largest between-group difference. During the follow-up period, the reduction in the insomnia subscale score tended to decrease, whereas reductions in the core and anxiety subscale scores were relatively maintained.

**Conclusion:**

Zuranolone showed consistent treatment effects regardless of baseline characteristics. During the treatment period, early improvement in depressive symptoms was accompanied by improvements across all symptom domains, and improvements in core and anxiety symptom domains appeared to be relatively maintained during the follow-up period. These symptom domain-specific temporal profiles could suggest patterns of antidepressant response and symptom change following treatment with zuranolone. Given the exploratory nature of this analysis, these findings should be interpreted descriptively and require confirmation in future hypothesis-driven studies.

## 1 Introduction

Major depressive disorder (MDD) is a severe psychiatric condition associated with high prevalence rates and substantial burden, including functional impairment, reduced quality of life, and elevated suicide risk (1). In Japan, the 12-month prevalence of MDD is reported to be 2.7%, with a lifetime prevalence of 5.7% (2). Despite the availability of various antidepressant medications, conventional treatments typically require several weeks to months before their therapeutic effects become apparent (3). This delayed onset of action leaves a critical gap in meeting patients’ needs for rapid symptom improvement (3, 4). When adequate symptom improvement is not achieved early in treatment, patients are at increased risk of persistent symptoms, disease progression, and difficulties with treatment adherence (5). Consequently, clinical practice urgently requires therapeutic options that can achieve prompt symptom relief (4). The development of faster-acting interventions could address this unmet need and potentially improve both short-term outcomes and long-term prognosis in patients with MDD.

Zuranolone is a neuroactive steroid that functions as a positive allosteric modulator of gamma-aminobutyric acid (GABA)-_A_ receptors, offering a novel mechanism of action that is distinct from that of conventional monoaminergic antidepressants (6, 7). Through this mechanism, neurosteroids may facilitate early improvement in depressive symptoms in patients with MDD (8, 9). Unlike traditional antidepressants that require chronic daily administration, zuranolone has been developed with a distinctive dosing regimen of 30 mg once daily for 14 consecutive days, offering an alternative treatment approach.

International studies and recent reviews of zuranolone have reported rapid and clinically meaningful improvements in depressive symptoms, further supporting the therapeutic potential of this mechanism of action (10–12, 25). However, despite these findings, zuranolone has not been approved by the U.S. Food and Drug Administration for the treatment of MDD.

Consistent with these international findings, clinical trials conducted in Japanese patients with MDD have demonstrated that zuranolone produces significant improvements in depressive symptoms as early as Day 3 of treatment, with sustained benefits observed through Day 15 (13, 14). This rapid onset of action represents a clinically meaningful advantage that addresses the delayed therapeutic response of existing antidepressants. Based on these positive findings from clinical trials in Japanese patients with MDD, zuranolone received manufacturing and marketing approval in Japan for the treatment of MDD in December 2025 (15).

The efficacy of zuranolone in Japanese patients with MDD has been evaluated in Phase 2 and Phase 3 double-blind, randomized, placebo-controlled trials (13, 14). In both studies, zuranolone 30 mg administered once daily for 14 days demonstrated a statistically significant improvement compared to placebo in the primary endpoint of change from baseline in the 17-item Hamilton Rating Scale for Depression (HAM-D17) total score on Day 15. The most common adverse events included somnolence and dizziness; however, no serious safety signals were identified in either trial. These findings indicate that zuranolone may be beneficial in improving symptoms in the early phase of depression treatment in Japanese patients with MDD, with a safety profile characterized by mild-to-moderate adverse events and no major safety concerns.

The Phase 2 trial showed a significant reduction in the HAM-D17 insomnia subscale score on Day 15, whereas reductions in the core and anxiety subscale scores, although numerically favorable, did not reach statistical significance (14). In the Phase 3 trial, the Insomnia Severity Index (ISI) total scores were significantly lower on Day 3, Day 8, and Day 15 in the zuranolone 30 mg group than in the placebo group (13), further suggesting that zuranolone may exert particularly notable effects on sleep-related domains. While these clinical trials demonstrated improvements in depressive symptoms with zuranolone treatment, several unresolved clinical questions remain regarding a more refined understanding of its therapeutic effect. The primary unresolved question is how changes in specific symptom domains—core, anxiety, and insomnia—are associated with the early reduction in the HAM-D17 total score and how these symptom domains evolve during and after the treatment period. Although the mean HAM-D17 total score was maintained at the group level during the follow-up period, recent trajectory pattern analyses have shown that a subset of patients experiences an increase in the HAM-D17 total score immediately after the end of the 14-day treatment period, predominantly driven by worsening insomnia symptoms (16). These findings highlight the importance of examining symptom-specific trajectories rather than relying solely on aggregate scores to better understand the temporal dynamics of treatment response. In MDD, core symptoms such as depressed mood and anhedonia represent the central features of the disorder; however, anxiety and sleep disturbances frequently co-occur with these core symptoms and often persist as residual symptoms after treatment. Importantly, these non-core symptom domains have been shown to adversely affect recovery, relapse risk, and functional outcomes (17–19). Therefore, characterizing the contribution and temporal pattern of improvement across individual symptom domains is of substantial clinical relevance, beyond the overall improvement in depressive symptoms. In addition, understanding whether treatment effects are consistent across patient subgroups defined by baseline characteristics, such as age, sex, and baseline symptom severity, is clinically relevant for interpreting the generalizability of these effects. However, because individual clinical trials are primarily powered to evaluate predefined primary endpoints, they often lack sufficient statistical power for detailed symptom domain-specific or subgroup analyses, leaving uncertainty regarding treatment effect heterogeneity.

We hypothesized that zuranolone would demonstrate greater reductions in HAM-D17 total scores than placebo and that these treatment effects would be generally consistent across major baseline patient characteristics. Given its distinct mechanism of action as a neuroactive steroid and positive allosteric modulator of GABA_A_ receptors, we further hypothesized that the antidepressant effects of zuranolone would not be limited to improvements in insomnia symptoms but would also involve improvements in core and anxiety symptom domains, resulting in distinct temporal patterns across HAM-D17 symptom domains.

To address these unresolved questions and provide a more comprehensive characterization of the antidepressant effects of zuranolone in Japanese patients with MDD, we conducted a pooled analysis integrating data from the Phase 2 and Phase 3 trials of zuranolone 30 mg and placebo groups. By pooling data from these studies, this analysis enhances the statistical power and precision of effect estimates, which are often limited in individual trials. Given the *post hoc* nature of this pooled analysis, the analyses were exploratory and hypothesis-generating, and the results should be interpreted descriptively. The pooled analysis was conducted with three primary objectives. First, we evaluated the temporal trajectory of the HAM-D17 total score throughout the treatment and follow-up periods to characterize both the onset of efficacy and the pattern of symptom evolution. Second, we analyzed the treatment effects across key baseline characteristics, including age, sex, and baseline symptom severity, to investigate the potential heterogeneity in the response to treatment. Third, we investigated the relationship between overall antidepressant efficacy and improvements in specific symptom domains by analyzing the HAM-D17 subscale scores corresponding to core, anxiety, and insomnia. Together, these analyses aim to refine the therapeutic profile of zuranolone and provide clinically meaningful insights to inform treatment decision-making for MDD.

## 2 Materials and methods

### 2.1 Study design and data source

This *post hoc* pooled analysis of two randomized, double-blind, placebo-controlled trials conducted in Japan assessed the efficacy and safety of zuranolone in adults with MDD. In the Phase 2 trial (jRCT2080225180), participants were randomized in a 1:1:1 ratio to receive either zuranolone 20 mg, zuranolone 30 mg, or placebo, administered orally once daily for 14 days (14). Following the 14-day treatment period, a 6-week follow-up without the study drug was conducted to evaluate the durability of the treatment response. The Phase 3 trial (jRCT2031210577) comprised two consecutive parts. In Part A, participants were randomized in a 1:1 ratio to receive either zuranolone 30 mg or placebo, administered orally once daily for 14 days, followed by a 6-week follow-up period (13). Part B was an open-label extension for participants who completed Part A, permitting repeated treatment cycles following the same procedures as in Part A, as clinically indicated (20). This *post hoc* analysis utilized data from the zuranolone 30 mg and placebo groups of the Phase 2 trial and Part A of the Phase 3 trial to conduct a longitudinal assessment of symptom changes throughout the treatment and follow-up periods. In both the Phase 2 and Phase 3 trials, the primary endpoint was the change from baseline in the HAM-D17 total score on Day 15.

### 2.2 Participants

The majority of the eligibility criteria for the Phase 2 and Phase 3 trials were the same. Eligible participants were Japanese adults aged 18–75 years who met the Diagnostic and Statistical Manual of Mental Disorders, 5th edition (DSM-5) criteria for MDD, with a current MDD episode ongoing for at least 8 weeks but no more than 12 months prior to signing the informed consent form (ICF). Participants were also required to have a HAM-D17 total score of ≥22 and a Patient Health Questionnaire-9 (PHQ-9) score of ≥15 on Day 1 (baseline/visit 1). The key exclusion criteria included serious comorbid medical conditions, treatment-resistant depression (TRD; no improvement in depressive symptoms with at least two different antidepressants [excluding antipsychotics] for an existing depressive episode at adequate approved doses for 4 weeks, and using the Massachusetts General Hospital Antidepressant Treatment Response Questionnaire), use of antidepressants within 14 days prior to Day 1, and risk of suicide.

In the Phase 2 trial, 250 participants were randomized to receive placebo (n = 83), zuranolone 20 mg (n = 85), or zuranolone 30 mg (n = 82), whereas 412 participants were randomized to receive either placebo (n = 205) or zuranolone 30 mg (n = 207) in the Phase 3 trial. The demographics and baseline characteristics of the participants in the Phase 2 and Phase 3 trials were similar; therefore, it was deemed reasonable to conduct a pooled analysis.

The efficacy analyses utilized the full analysis set (FAS), which encompassed all participants who received at least one dose of either zuranolone 30 mg or a placebo and had HAM-D17 assessments available at baseline and at least one subsequent assessment time point. The safety analysis set included all participants who received at least one dose of either zuranolone 30 mg or a placebo and had at least one post-dose safety assessment.

### 2.3 Efficacy analysis

The evaluated outcomes included changes from baseline in the HAM-D17 total score and subscale scores. In addition, changes from baseline in the HAM-D17 total score stratified by baseline patient characteristics were assessed. The core subscale consists of depressed mood, feeling of guilt, suicide, work and activities, and retardation (21). The anxiety subscale consists of anxiety psychic, anxiety somatic, somatic symptoms gastrointestinal, somatic symptoms general, hypochondriasis, and insight (22). The insomnia subscale consists of insomnia early, insomnia middle, and insomnia late (23). The other symptom subscale consists of agitation, genital symptoms, and loss of weight. The relative contributions of each subscale score to the difference between the zuranolone and placebo groups in the change from baseline in the HAM-D17 total score were also examined. Other outcomes included treatment response, defined as ≥50% reduction from baseline in the HAM-D17 total score; remission, defined as a HAM-D17 total score ≤7; individual HAM-D17 item scores; and response of Clinical Global Impression of Improvement (CGI-I) score, defined as “much improved” or “very much improved,” and the ISI assessed throughout the study period.

The changes from baseline to each time point between Day 3 and Day 57 in the HAM-D17 total score were analyzed to compare the zuranolone and placebo groups using a mixed-effects model for repeated measures (MMRM). This analysis utilized all available data collected from Day 3 to Day 57. The MMRM was employed with the change from baseline in the HAM-D17 total score as a response variable. The model included the study identifier, intervention group, time point, and the interaction between the intervention group and time point as fixed effects, whereas the HAM-D17 total score at baseline, the presence or absence of prior medication for depressive episodes, and sex were incorporated as covariates in the model with unstructured covariance matrix. The Kenward-Roger method was used for adjusting degrees of freedom. The SAS code used for the MMRM analysis described above is provided in **Supplementary Method 1**. Similar analyses were conducted for the patient subgroups. Additionally, analogous analyses were performed using the core, anxiety, insomnia, and other symptom subscale scores of the HAM-D17, individual HAM-D17 item scores, ISI total score, and individual ISI scores as response variables, incorporating the corresponding baseline scores as covariates in each model. To assess the antidepressant efficacy of zuranolone and the relative contributions of improvements across symptom domains, the relative contribution of each subscale score to the difference between the zuranolone and placebo groups in change from baseline in the HAM-D17 total score was calculated from Day 3 to Day 57.

For binary endpoints, the inverse probability-weighted generalized estimating equation (IPW-GEE) model was used to construct odds ratios to compare the HAM-D17 response and remission and the response of the CGI-I score. Using all available data obtained from Day 3 to Day 57, the IPW-GEE model was applied, with the study identifier, intervention group, time point, and interaction between the intervention group and time point as fixed effects, and the score at baseline, presence or absence of prior drug treatment for depressive episodes, and sex as covariates. The IPW-GEE model assumes an independent structure for the working correlation matrices. When the model did not converge owing to a small number of events, data at the earliest time points (Day 3) were excluded from the IPW-GEE model. All analyses were *post hoc* in nature, and a two-sided P-value < 0.05 was used as the threshold for statistical significance in this study. Because no adjustments for multiple comparisons were performed, all reported P-values should be considered nominal and interpreted descriptively.

### 2.4 Ethics statement

This pooled *post hoc* analysis was conducted using data from two previously approved Phase 2 and Phase 3 clinical trials. All original clinical trials were conducted in accordance with the Declaration of Helsinki, ICH-GCP guidelines, and applicable local regulations, and were approved by the institutional review boards or ethics committees at each study site. Written informed consent was obtained from all participants prior to participation in the original clinical trials. Based on the nature of this secondary analysis using existing clinical trial data, additional ethical approval or informed consent was not sought.

### 2.5 Safety analysis

Adverse events (AEs) were categorized by System Organ Class and Preferred Terms according to the Medical Dictionary for Regulatory Activities (MedDRA) Version 24.1. Among the AEs documented in the electronic case report forms, treatment-emergent adverse events (TEAEs) were incorporated into the safety analyses. TEAE was defined as any AE with an onset date on or after the administration of the initial dose of the study drug. The most common TEAEs with an incidence of ≥5% in the zuranolone group during the study period are tabulated. All analyses were conducted using SAS version 9.4.

## 3 Results

### 3.1 Patient disposition

The pooled FAS/safety analysis set from both studies included 284 participants in the zuranolone group (79 from the Phase 2 trial and 205 from the Phase 3 trial) and 278 participants in the placebo group (79 from the Phase 2 trial and 199 from the Phase 3 trial) (**Supplementary Figure S1**).

### 3.2 Demographics and baseline characteristics

The baseline demographics and clinical characteristics were well-balanced across the treatment groups (**Table 1**). The mean (SD) age was 39.5 (12.5) years in the zuranolone group and 40.6 (11.9) years in the placebo group. Baseline HAM-D17 total scores were comparable between groups, with mean (SD) values of 24.3 (2.1) in the zuranolone group and 24.2 (2.1) in the placebo group.

**Table 1:** Demographics and baseline characteristics (full analysis set).

Characteristic	Category	Zuranolone 30 mg N = 284 n (%)	Placebo N = 278 n (%)
Sex	Male	136 (47.9)	132 (47.5)
	Female	148 (52.1)	146 (52.5)
Age (years)	Mean (SD)	39.5 (12.5)	40.6 (11.9)
	≥18 to <25	42 (14.8)	28 (10.1)
	≥25 to <45	142 (50.0)	138 (49.6)
	≥45 to <65	94 (33.1)	106 (38.1)
	≥65	6 (2.1)	6 (2.2)
BMI (kg/m^2^)	Mean (SD)	22.83 (4.55)	23.25 (4.47)
Baseline value of HAM-D17 total score	Mean (SD)	24.3 (2.1)	24.2 (2.1)
	≥22 to ≤24	180 (63.4)	175 (62.9)
	≥25	104 (36.6)	103 (37.1)
Classification based on DSM-5	Single episode	120 (42.3)	118 (42.4)
	Recurrent	164 (57.7)	160 (57.6)
Episode recurrences	1st time	121 (42.6)	117 (42.1)
	2nd time	90 (31.7)	92 (33.1)
	3rd-7th time	69 (24.3)	67 (24.1)
	No less than 8 times	2 (0.7)	1 (0.4)
	Unknown	2 (0.7)	1 (0.4)
Duration of current episode at randomization (months)^[a]^	2-4	92 (32.4)	97 (34.9)
	4-6	69 (24.3)	59 (21.2)
	6-8	50 (17.6)	54 (19.4)
	8-10	32 (11.3)	32 (11.5)
	10-12	31 (10.9)	32 (11.5)
	12 or more	10 (3.5)	4 (1.4)
Presence or absence of prior therapy for depressive episodes	Yes	0	0
	No	284 (100.0)	278 (100.0)
Presence or absence of prior drug for depressive episodes	Yes	158 (55.6)	155 (55.8)
	No	126 (44.4)	123 (44.2)

All data are expressed as n (%) unless speciﬁed. Abbreviations: BMI, body mass index; DSM-5, Diagnostic and Statistical Manual of Mental Disorders, ﬁfth edition; HAM-D17, 17-item Hamilton Rating Scale for Depression; MDD, major depressive disorder; SD, standard deviation.

[a] Duration of the current episode at randomization = (Date of randomization) – (Onset date) + 1.

### 3.3 Efficacy

The MMRM analysis of changes from baseline in HAM-D17 total score from Day 3 to Day 57 converged successfully and demonstrated a significant improvement in fit compared with the null model (likelihood ratio test: χ² = 7088.67, df = 44, P < 0.0001). In the MMRM analysis, significant effects were observed for study intervention (P = 0.0092), time point (P < 0.0001), and the interaction between study intervention and time point (P = 0.0233), indicating that the trajectory of HAM-D17 total score differed between the intervention groups over time (**Supplementary Table S1**).

Zuranolone showed a significantly greater reduction in the HAM-D17 total score compared with placebo on Day 3 (adjusted mean difference (aMD): −1.40; 95% CI: −1.95, −0.86; P < 0.0001), Day 8 (aMD: −1.86; 95% CI: −2.62, −1.09; P < 0.0001), and Day 15 (aMD: −1.48; 95% CI: −2.43, −0.53; P = 0.0023) (**Figure 1**). During the follow-up period (Day 15 to Day 57), the reductions in the HAM-D17 total score observed during the treatment period were maintained in both groups, and the between-group differences persisted numerically throughout the follow-up period.

**Figure 1:**
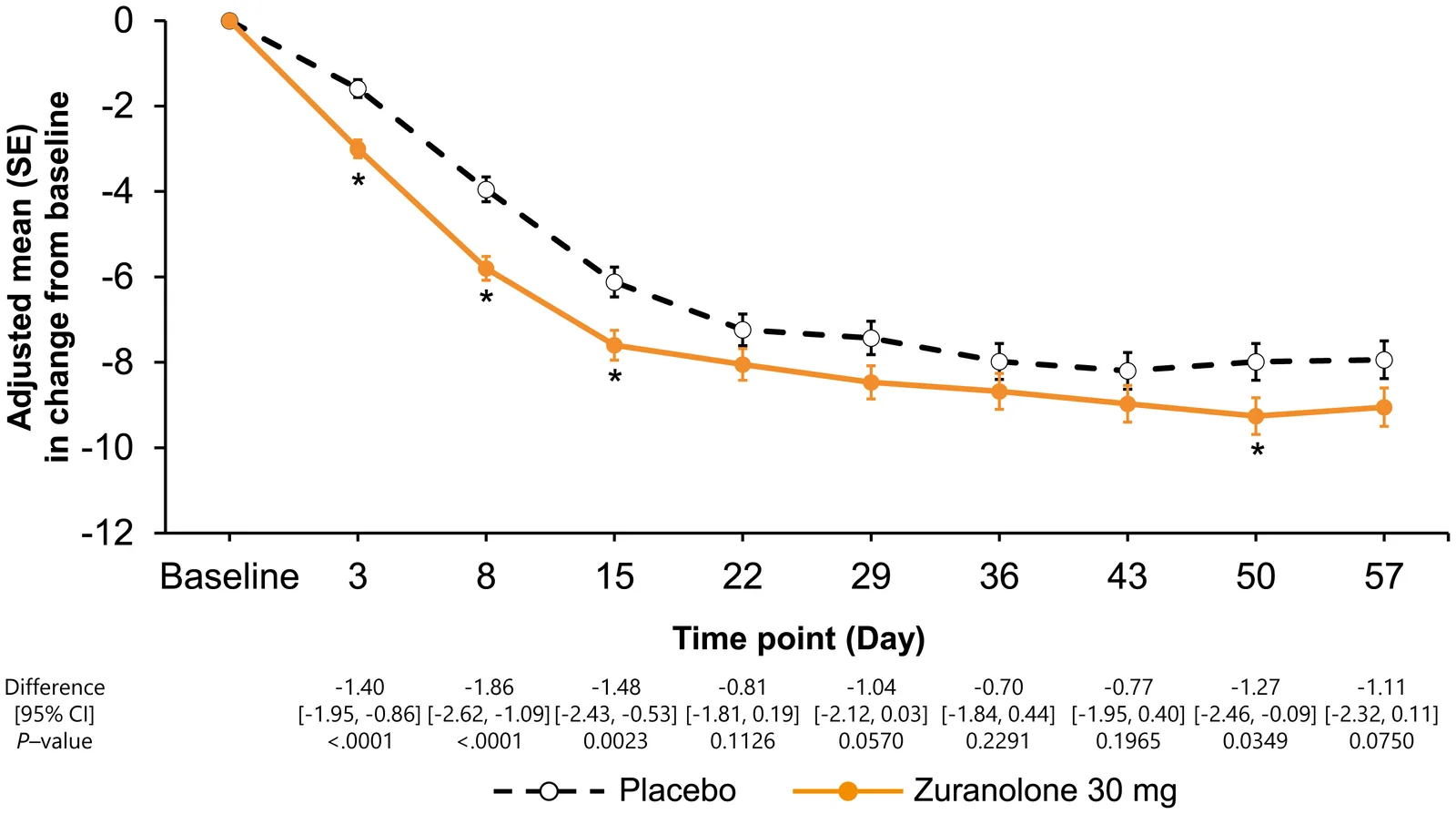
Adjusted mean (SE) change from baseline in the HAM-D17 total score over 57 days in the zuranolone 30 mg group compared with the placebo group. A mixed-effects model for repeated measures with unstructured covariance structure was applied to estimate the treatment difference between zuranolone and placebo. Change from baseline was the response variable; study intervention, study identifier, time point and interaction between study intervention and time point were included as fixed effects, with HAM-D17 total score at baseline, presence or absence of prior drug for depressive episodes, and sex as covariates. *P < 0.05 (Nominal P-values are presented without adjustment for multiple comparisons and should be interpreted descriptively and as hypothesis-generating). Abbreviations: CI, confidence interval; HAM-D17, 17-item Hamilton Rating Scale for Depression; SE, standard error.

Participants receiving zuranolone had significantly higher response rates (defined as ≥50% reduction from baseline in the HAM-D17 total score) on Day 3 (adjusted odds ratio (aOR): 5.11; 95% CI: 1.11, 23.64; P = 0.0368), Day 8 (aOR: 2.67; 95% CI: 1.47, 4.84; P = 0.0012), and Day 15 (aOR: 1.64; 95% CI: 1.07, 2.50; P = 0.0227) than those receiving placebo (**Supplementary Figure S2A**). Although no statistically significant differences between the placebo and zuranolone groups were observed during the follow-up period, the response rates were maintained in both groups, with consistently higher rates in the zuranolone group than in the placebo group. The zuranolone group showed a significantly higher remission rate (defined as HAM-D17 total score ≤7) than the placebo group on Day 8 (aOR: 5.16; 95% CI: 1.12, 23.85; P = 0.0355); however, on Day 15, the difference in remission rates between the zuranolone and placebo groups did not reach statistical significance (aOR: 1.94; 95% CI: 0.94, 4.00; P = 0.0741) (**Supplementary Figure S2B**). During the follow-up period, the remission rates in the zuranolone 30 mg group remained consistently higher than those in the placebo group.

Changes from baseline in the HAM-D17 total score on Day 15 were examined across subgroups using forest plots (**Figure 2**). The treatment effects were consistent across baseline patient characteristics, with point estimates indicating greater reductions in the HAM-D17 total score in the zuranolone group compared with the placebo group.

**Figure 2:**
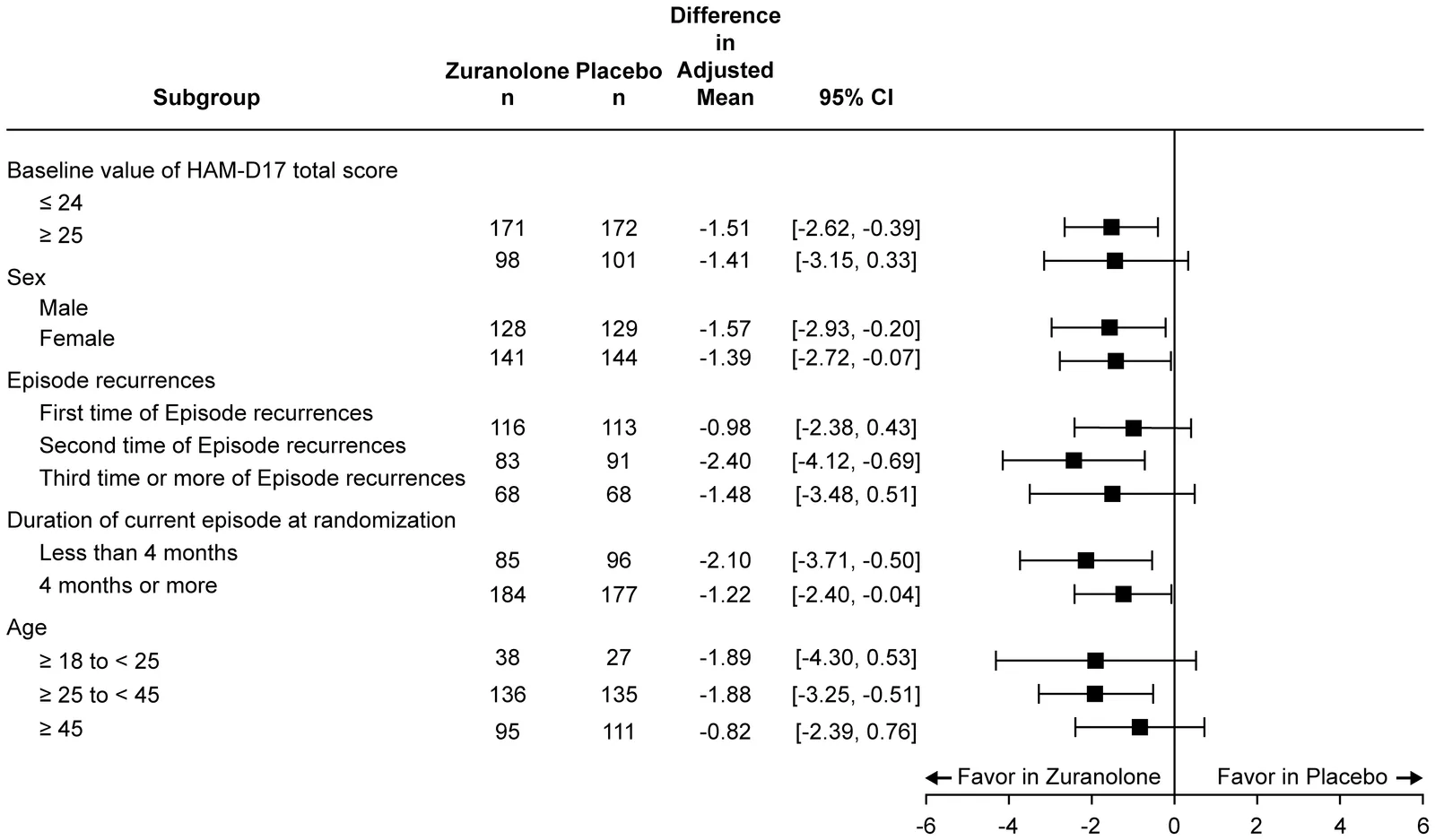
Forest plot of subgroup analyses showing change from baseline in HAM-D17 total score at Day 15 (full analysis set) between the zuranolone 30 mg and placebo groups. CI, confidence interval; HAM-D17, 17-item Hamilton Rating Scale for Depression.

For the HAM-D17 core subscale score, the zuranolone group showed significantly greater reductions than the placebo group on Day 3 (aMD: −0.37; 95% CI: −0.59, −0.15; P = 0.0011) and Day 8 (aMD: −0.34; 95% CI: −0.68, 0.00; P = 0.0479). On Day 15, the mean change from baseline was numerically greater in the zuranolone group than in the placebo group (aMD: −0.22; 95% CI: −0.64, 0.21; P = 0.3153) (**Figure 3A**). Regarding the HAM-D17 anxiety subscale score, the zuranolone group consistently showed reductions compared to the placebo group during the treatment period, with statistically significant between-group differences observed on Day 3 (aMD: −0.25; 95% CI: −0.47, −0.03; P = 0.0238), Day 8 (aMD: −0.51; 95% CI: −0.81, −0.22; P = 0.0007), and Day 15 (aMD: −0.36; 95% CI: −0.71, −0.01; P = 0.0455) (**Figure 3B**). For the HAM-D17 insomnia subscale score, the zuranolone group showed significantly greater reductions than the placebo group during the treatment period, with statistically significant between-group differences on Day 3 (aMD: −0.78; 95% CI: −1.02, −0.55; P < 0.0001), Day 8 (aMD: −0.82; 95% CI: −1.08, −0.56; P < 0.0001), and Day 15 (aMD: −0.75; 95% CI: −1.02, −0.48; P < 0.0001) (**Figure 3C**). After the treatment period, worsening of the insomnia subscale scores was observed in the zuranolone group, resulting in an attenuation of the between-group difference compared with the placebo group. In contrast, the core and anxiety subscale scores did not show a clear worsening in either group during the follow-up period, and the between-group differences were generally maintained. Further details at the symptom level were examined in the forest plot, showing changes from baseline in each HAM-D17 item score (**Supplementary Figure S3**).

**Figure 3:**
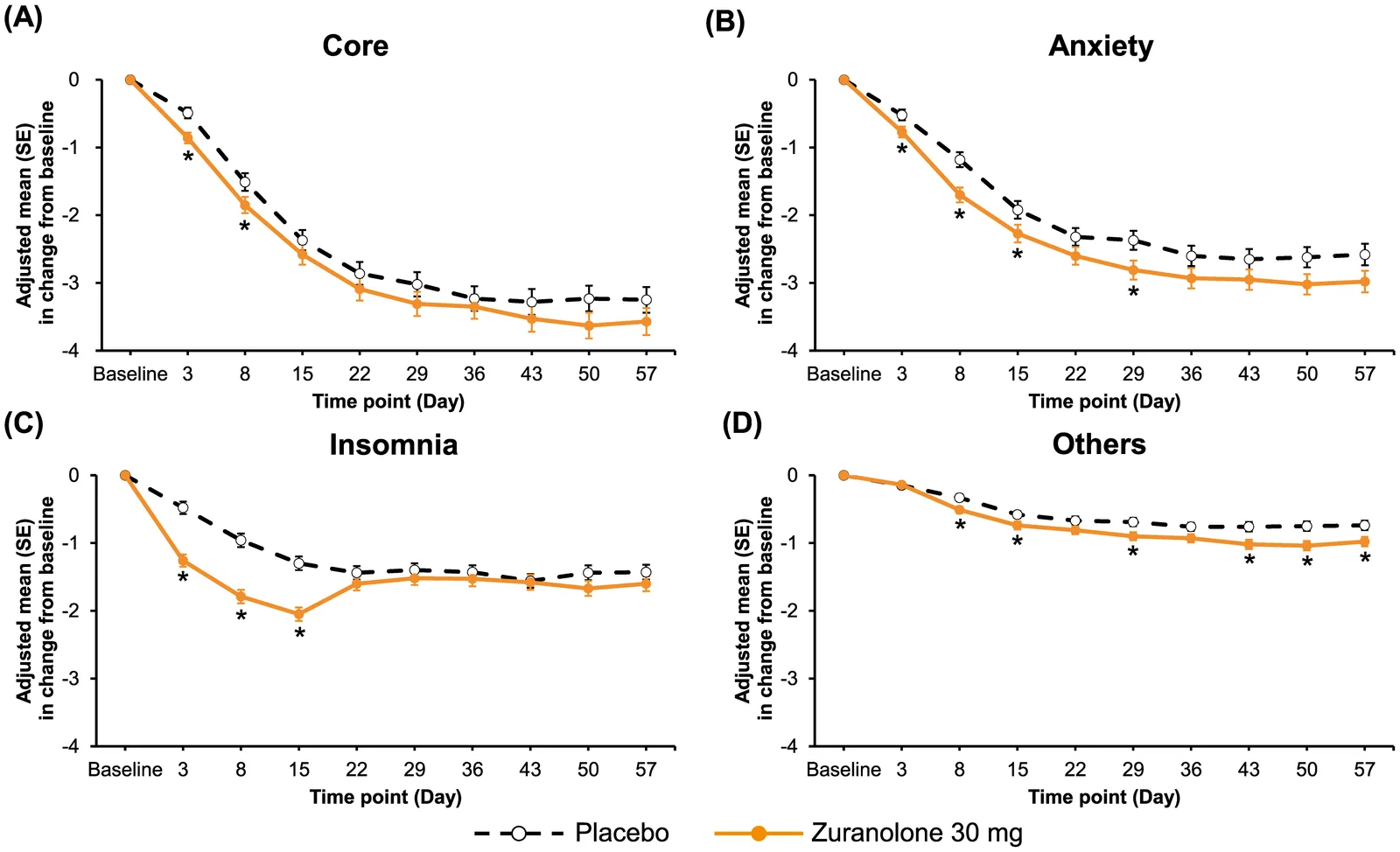
Adjusted mean (SE) change from baseline in **(A)** the core subscale score **(B)** the anxiety subscale score **(C)** the insomnia subscale score and **(D)** the others subscale score over 57 days in the zuranolone 30 mg group compared with the placebo group. A mixed-effects model for repeated measures with unstructured covariance structure was applied to estimate treatment difference between zuranolone 30 mg and placebo; change from baseline was the response variable; study intervention, study identifier, time point, and interaction between study intervention and time point were included as fixed effects, with subscale score at baseline, presence or absence of prior drug for depressive episodes, and sex as covariates. *P < 0.05 (Nominal P-values are presented without adjustment for multiple comparisons and should be interpreted descriptively and as hypothesis-generating). HAM-D17, 17-item Hamilton Rating Scale for Depression; SE, standard error.

The relative contributions of the HAM-D17 subscales to the difference between the zuranolone and placebo groups in changes from baseline in the HAM-D17 total score were evaluated at each time point. During the treatment period, the insomnia subscale accounted for the largest proportion of the overall treatment difference; however, its relative contribution decreased during the follow-up period (**Figure 4**). In contrast, the core and anxiety subscales showed consistent contributions during the treatment period and accounted for more than 50% of the difference at each time point during the follow-up period.

**Figure 4:**
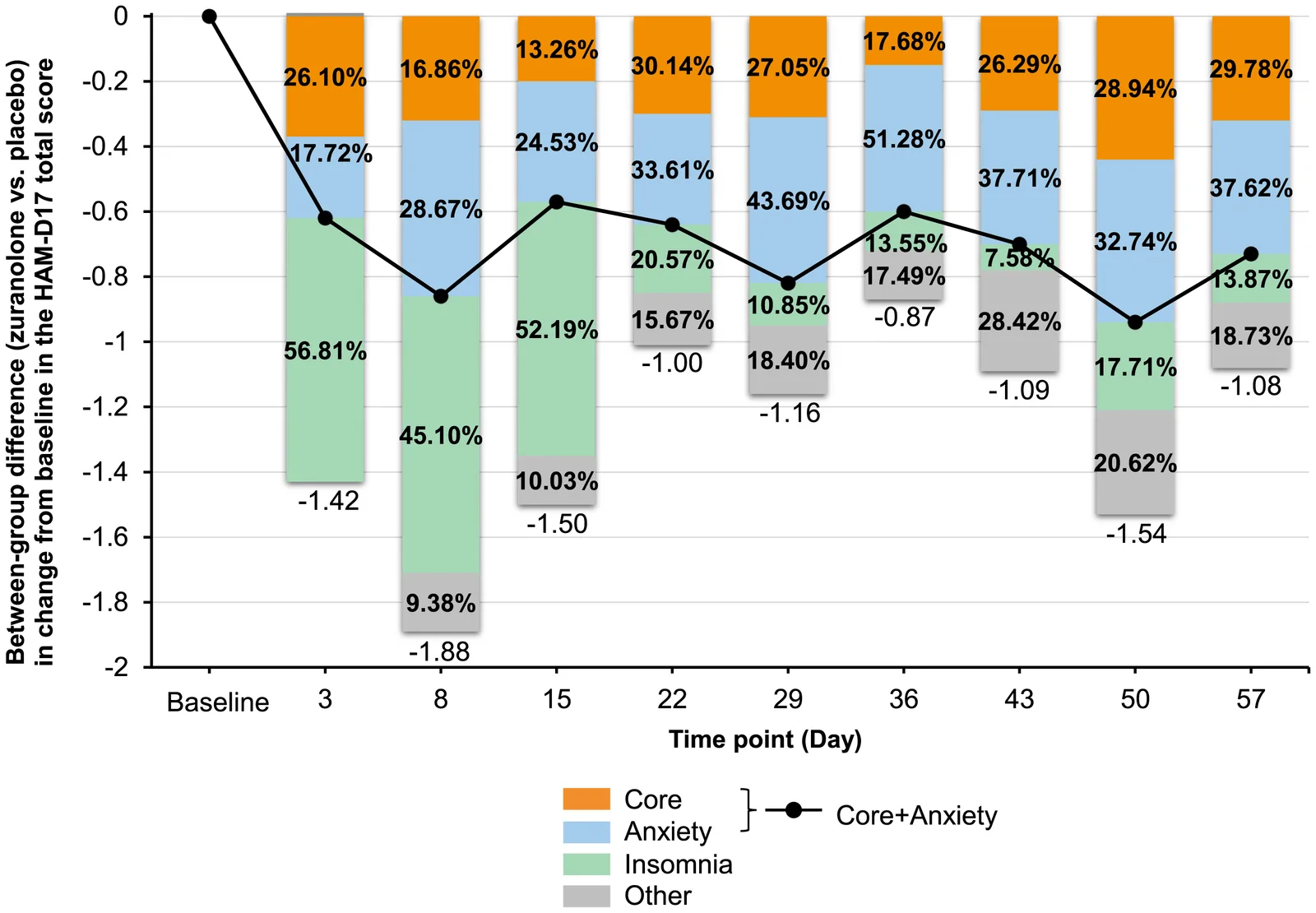
Relative proportion of each subscale score contributing to the between-group difference (zuranolone vs. placebo) in change from baseline in the HAM-D17 total score. HAM-D17, 17-item Hamilton Rating Scale for Depression Relative proportion of each subscale score was calculated by dividing the difference in the mean of each subscale score by the difference in the mean of the HAM-D17 total scores.

Based on physician-rated CGI-I scores, the response rate (defined as “much improved” or “very much improved”) was significantly higher in the zuranolone group than in the placebo group on Day 3 (aOR: 4.62; 95% CI: 1.54, 13.79; P = 0.0062) and Day 8 (aOR: 2.99; 95% CI: 1.77, 5.04; P < 0.0001) (**Supplementary Figure S4**). After the 14-day treatment period, the response rate using the CGI-I remained higher in the zuranolone group than in the placebo group at all time points through Day 57; however, the difference was not significant between groups. The ISI total scores were significantly lower on Day 3, Day 8, and Day 15 in the zuranolone group than in the placebo group, with statistically significant adjusted mean between-group differences observed on Day 3 (aMD: -0.87, 95% CI: -1.38, -0.36; P = 0.0009), Day 8 (aMD: -1.33, 95% CI: -2.03, -0.63; P = 0.0002), and Day 15 (aMD: -1.57, 95% CI: -2.41, -0.73; P = 0.0003) (**Supplementary Figure S5**).

### 3.4 Safety

No deaths or serious TEAEs were reported during the study period in the Phase 2 and Phase 3 trials. TEAEs leading to discontinuation of the study drug were reported in 2 participants (5 events) in the zuranolone group alone. When categorized into the 14-day treatment and follow-up periods, TEAEs were observed in 45.1% and 26.8% of patients in the zuranolone group and 25.9% and 26.3% of patients in the placebo group, respectively. The most common TEAEs occurring with an incidence of ≥5% in the zuranolone group during the treatment period were somnolence (zuranolone group, 15.5%; placebo group, 5.4%) and dizziness (zuranolone group, 11.3%; placebo group, 1.4%) (**Table 2**). Overall, all TEAEs were non-severe, and most participants experienced mild or moderate TEAEs. The rate of treatment discontinuation owing to TEAEs was low, and no new safety signals were observed (**Table 2**).

**Table 2:** Incidence of TEAEs by severity and TEAEs in ≥ 5% of participants during the treatment and follow-up periods (safety analysis set).

System organ class^†^ - preferred term	Zuranolone 30 mg (*N* = 284)		Placebo (*N* = 278)	
	Treatment period *n* (%)	Follow-up period *n* (%)	Treatment period *n* (%)	Follow-up period *n* (%)
Participants with any TEAE	128 (45.1)	76 (26.8)	72 (25.9)	73 (26.3)
Severity				
Mild	111 (39.1)	60 (21.1)	65 (23.4)	61 (21.9)
Moderate	17 (6.0)	16 (5.6)	7 (2.5)	12 (4.3)
Severe	0	0	0	0
TEAEs in ≥5% of participants				
Somnolence	44 (15.5)	0	15 (5.4)	2 (0.7)
Dizziness	32 (11.3)	1 (0.4)	4 (1.4)	2 (0.7)

Treatment period: up to 2 weeks; follow-up period: 2–8 weeks.

The definition of N used in the follow-up period was the same as that used in the treatment period.

AEs that occurred after discontinuation in the treatment period were counted as events in the follow-up period.

AE, adverse event; MedDRA, Medical Dictionary for Regulatory Activities; TEAE, treatment-emergent adverse event.

† System Organ Class and Preferred Term as per MedDRA version 24.1.

## 4 Discussion

This study presents a pooled analysis of Phase 2 and Phase 3 data evaluating the efficacy and safety of zuranolone 30 mg administered over a 14-day treatment period in Japanese patients with MDD. This comprehensive approach allows for a detailed understanding of the treatment effect profile across multiple dimensions, considering total symptoms, patient demographics and symptom domains. Statistically significant reductions in the HAM-D17 total score were observed in the zuranolone group compared with the placebo group during the treatment period; however, there was no significant difference between the zuranolone and placebo groups during the follow-up period (except on Day 50). The treatment effects were consistent across all subgroups, with zuranolone demonstrating superior efficacy compared to placebo. During the treatment period, zuranolone was associated with reductions in the HAM-D17 core, anxiety, and insomnia subscale scores. However, during the follow-up period, reductions in the insomnia subscale score tended to decrease, whereas reductions in the core and anxiety subscale scores were generally maintained after the end of the treatment period.

Analyses stratified by major demographic factors, such as age, sex, and baseline symptom severity, showed generally consistent reductions in the HAM-D17 total scores within the evaluated range. Given that sex differences in endogenous neurosteroids (allopregnanolone) have been reported, it is theoretically plausible that the antidepressant effect of zuranolone may also exhibit sex differences (24), potentially more pronounced in females. However, this subgroup analysis did not identify any clear differences in treatment effect by sex; both males and females exhibited greater reductions in HAM-D17 total scores compared with placebo, suggesting that zuranolone may also be effective in male patients. In US MDD trials, even in studies where the primary endpoint was not achieved in the overall population (such as the MOUNTAIN trial in the US, zuranolone 20/30 mg), *post hoc* analysis suggested a more pronounced effect of 30 mg zuranolone in the subgroup of patients with higher severity (HAM-D17 ≥24) (25). However, in this analysis, the treatment effect was observed regardless of the baseline HAM-D17 severity. It is important to note that since the Japanese Phase 2 and Phase 3 trials enrolled only patients with HAM-D17 scores of 22 or higher, evaluating the effect of zuranolone in patients with milder depression is warranted in future studies. Taken together, these findings suggest that within the range of patient demographics evaluated in these clinical trials, the effect of zuranolone may not be confined to specific subpopulations.

The analysis of symptom domains showed relatively greater reductions in the insomnia subscale score during the treatment period, and statistically significant reductions during the treatment period were also observed in both the core and anxiety subscale scores. Sleep symptoms play a crucial role in the treatment of depression, and interventions targeting insomnia have been reported to be associated with improvements in depressive symptoms beyond insomnia itself (26, 27). During the follow-up period, reductions in the insomnia subscale score tended to decrease, whereas reductions in the core and anxiety subscale scores remained relatively stable, comparable to those observed at the end of treatment. Furthermore, a contribution analysis of the difference between the zuranolone and placebo groups in the HAM-D17 total score indicated that during the treatment period, the reduction in the insomnia subscale score accounted for the largest between-group difference, whereas during the follow-up period, reductions in the core and anxiety subscale scores accounted for a substantial proportion of the observed between-group difference. Collectively, these findings could suggest improvements across the insomnia, core, and anxiety symptom domains with zuranolone. However, because depressive symptoms are highly interconnected, it remains unclear whether the observed improvements in core and anxiety symptoms reflect direct effects on these domains or secondary changes associated with improvements in insomnia symptoms. Given the exploratory nature of this analysis, these findings should be interpreted descriptively and require confirmation in future hypothesis-driven studies.

From a mechanistic perspective, the observed symptom domain-specific temporal profiles may be compatible with the pharmacological properties of neuroactive steroids and the GABAergic deficit hypothesis of depression (28). Chronic stress reduces endogenous allopregnanolone levels, thereby reinforcing maladaptive excitatory states and contributing to the disruption of excitation/inhibition balance associated with depressive symptoms (28, 29). Neurosteroids, including allopregnanolone and its synthetic analogue zuranolone, positively modulate GABA_A_ receptors and potentiate phasic and tonic inhibition via the activation of synaptic and extrasynaptic GABA_A_ receptors, respectively (28, 30). Unlike benzodiazepines, which primarily target synaptic GABA_A_ receptors and show limited antidepressant efficacy, such broader enhancement of GABAergic transmission has been proposed to affect core depressive symptom domains by facilitating recovery and stabilization of glutamatergic and GABAergic synaptic function, normalizing the excitation/inhibition imbalance, and potentially supporting the persistence of these effects beyond the treatment period (28). In contrast, synaptic GABA_A_ receptor modulation, as typified by benzodiazepines, is associated with rapid and potent enhancement of GABAergic neurotransmission and hypnotic effects; however, these effects are generally transient and may diminish over time (31). In this context, the worsening of insomnia subscale scores after the treatment period with zuranolone may be related to the attenuation of hypnotic effects via synaptic GABA_A_ receptor modulation. Importantly, the observed worsening appeared to represent a partial loss of treatment-period improvement and a return toward baseline severity rather than worsening beyond pretreatment baseline severity. Therefore, the present findings do not provide clear evidence of rebound insomnia. However, because this pooled *post hoc* analysis was not specifically designed to evaluate rebound insomnia or withdrawal-related phenomena, further studies incorporating mechanistic endpoints are required to clarify the biological basis of these clinical findings.

Core symptoms have been reported to be closely linked to overall depression severity and are associated with functional impairment and reduced quality of life; therefore, early improvement in core symptoms is a critical clinical goal (32, 33). Anxiety symptoms are frequent comorbidities in depression and are key clinical factors that can exacerbate treatment outcomes; the STAR*D analysis has demonstrated that depression with anxiety is less likely to achieve remission and requires a longer duration for remission (34, 35). Moreover, even after remission in depression, residual symptoms such as anxiety and sleep disturbances frequently persist, exerting a strong impact on treatment outcomes (17, 19). Considering these clinical challenges, the observation that zuranolone contributes not only to reductions in the insomnia subscale score but also to reductions and sustained maintenance of the core and anxiety subscale scores may be clinically important.

Benzodiazepines that interact with GABA_A_ receptors are known to cause withdrawal symptoms, such as insomnia, anxiety, restlessness, headache, nausea, and vomiting (36). In the Phase 3 trial of zuranolone, AEs resembling withdrawal symptoms previously associated with benzodiazepine discontinuation were reported slightly more frequently in the zuranolone 30 mg group than in the placebo group during the follow-up period (13). Although the possibility that these events represent withdrawal symptoms cannot be definitively excluded, pooled analyses, including the Phase 2 trial, showed that the incidence of these events in the zuranolone group was ≤3%. In addition, the difference in incidence between the zuranolone and placebo groups was limited to 1–2% (headache, 1.4% [4/284] vs. 0.4% [1/278]; diarrhea, 2.1% [6/284] vs. 0.4% [1/278]; myalgia, 1.4% [4/284] vs. 0.4% [1/278]; and pyrexia, 2.5% [7/284] vs. 1.1% [3/278]).

This study had several limitations. It is important to note that this pooled analysis constitutes a *post hoc* exploratory investigation; no adjustments for multiplicity were conducted, and inter-study variability cannot be entirely excluded from the results of this study. Therefore, these aspects require careful consideration when interpreting results.

### 4.1 Conclusions

Consistent treatment effects of zuranolone were shown in Japanese patients with MDD across baseline characteristics, such as age, sex, and baseline severity, and zuranolone showed reductions in HAM-D17 scores across multiple symptom domains, including the core, anxiety, and insomnia subscales during the treatment period. However, during the follow-up period, reductions in the insomnia subscale score tended to decrease, whereas reductions in the core and anxiety subscale scores were relatively maintained. These findings could suggest that the effects of zuranolone across multiple symptom domains, beyond improvements in insomnia symptoms alone, may be associated with the overall amelioration of depressive symptoms and their subsequent trajectories in patients with MDD. Given the *post hoc* nature of this pooled analysis, these results should be interpreted descriptively and confirmed through hypothesis-driven research.

## Data Availability

Shionogi & Co., Ltd. is committed to disclosing the synopses and results of its clinical trials and sharing clinical trial data (raw dataset or study data tabulation model dataset) with researchers upon request. If the research proposal is reviewed and approved by an independent review panel, anonymized data and redacted documents will be provided in a secure research environment. For further details, please refer to the websites of Shionogi & Co., Ltd. (https://www.shionogi.com/global/en/company/policies/shionogi-group-clinical-trial-data-transparency-policy.html) and Vivli (https://vivli.org/).
