# Supplementary material for: Characterizing the efficacy of zuranolone across patient subgroups and symptom domains in Japanese patients with major depressive disorder: a pooled analysis of two placebo-controlled trials
Source: Front Psychiatry. 2026 Oct 22;17:1891881. doi: 10.3389/fpsyt.2026.1891881 (PMC13736616; doi:10.3389/fpsyt.2026.1891881)
Supplement: Supplementary file 1 [file Supplementaryfile1.docx]

Supplementary Material

# 1 Supplementary Method for the programming code

The SAS code used to perform the mixed-effects model for repeated measures (MMRM) analysis of change from baseline in HAM-D17 total score at each post-baseline visit between Day 3 and Day 57 is provided below.

proc mixed data = [input dataset] order = internal;

class TRT01PN AVISITN USUBJID SEX DRUG STUDYID;

model CHG = BASE SEX DRUG STUDYID TRT01PN AVISITN TRT01PN*AVISITN / ddfm = kr;

repeated AVISITN / type = un subject = USUBJID;

lsmeans TRT01PN*AVISITN / diff cl alpha = 0.05;

run;

TRT01PN: Intervention group (zuranolone, placebo)

CHG: Change from baseline in the HAM-D17 total score

BASE: HAM-D17 total score at baseline

USUBJID: Participant identifier

AVISITN: Time point for analysis (Day 3, Day 8, Day 15, Day 22, Day 29, Day 36, Day 43, Day 50, Day 57)

SEX: Sex (male, female)

DRUG: Presence or absence of prior drug for depressive episodes (Yes, No)

STUDYID: Study identifier (Phase 2, Phase 3)

# 2 Supplementary Figures

**Supplementary Figure 1.** Patient Disposition and Flow Through Phase 2 and Phase 3 Trials.

**Supplementary Figure 2.** Bar graph of (A) response and (B) remission by the HAM-D17 score over 57 days (full analysis set). IPW-GEE analysis. IPW-GEE with an independent structure for the working correlation matrix was applied to estimate treatment differences between the zuranolone 30 mg and placebo groups. Fixed-effects: study intervention, study identifier, time point, and interaction between study intervention and time point. Covariates: baseline value of the HAM-D17 total score, sex, and presence or absence of prior drug for depressive episodes. *P < 0.05 (Nominal P-values are presented without adjustment for multiple comparisons and should be interpreted descriptively and as hypothesis-generating). Response: The number of patients with nonmissing HAM-D17 total scores at the visit and at baseline was evaluated. The number of patients with a response was deﬁned as a ≥ 50% reduction in the baseline HAM-D17 total score. The denominator of the percentage is the number of patients with nonmissing HAM-D17 total score at the visit and at baseline. Remission: The number of patients with nonmissing HAM-D17 total scores at the visit was evaluated. The number of patients with remission was deﬁned as a HAM-D17 score ≤ 7. The denominator of the percentage was the number of patients with nonmissing HAM-D17 total score at the visit. In the analysis of remission, data on Day 3 were excluded from the IPW-GEE model to avoid nonconvergence. Abbreviations: CI, conﬁdence interval; HAM-D17, 17-item Hamilton Rating Scale for Depression; IPW-GEE, inverse probability-weighted generalized estimating equation.

**Supplementary Figure 3.** Forest plot for each HAM-D17 item between the zuranolone 30 mg and placebo groups in (A) the core subscale score, (B) the anxiety subscale score, (C) the insomnia subscale score, and (D) the others subscale score MMRM with unstructured covariance matrix was applied to estimate treatment differences between the zuranolone 30 mg and placebo groups. Fixed-effects: study intervention, study identifier, time point, and interaction between study intervention and time point. Covariates: baseline value of corresponding HAM-D item, sex, and presence or absence of prior drug for depressive episodes.

Abbreviations: CI, confidence interval; HAM-D17, 17-item Hamilton Rating Scale for Depression

**Supplementary Figure 4.** Bar graph of improvement by the CGI-I score over 57 days (full analysis set). IPW-GEE analysis. "Improvement" is defined as a rating of "Very much improved" or "Much improved". IPW-GEE was applied to estimate treatment difference between zuranolone and placebo; study intervention, study identifier, time point, and interaction between study intervention and time point were included as fixed effects, with the CGI-S score at baseline, presence or absence of prior drug for depressive episodes, and sex as covariates. IPW-GEE assumed an independent structure for the working correlation matrix. *P < 0.05 (Nominal P-values are presented without adjustment for multiple comparisons and should be interpreted descriptively and as hypothesis-generating).

If the model was not converged, the analysis time points were excluded in order of early time points until the model was converged, and '---' was shown at the excluded time points. Abbreviations: CI, confidence interval; CGI-I, Clinical Global Impression - Global Improvement; CGI-S, Clinical Global Impression - Severity of Illness

**Supplementary Figure 5.** (A) Adjusted mean (SE) change from baseline in ISI total score over 57 days in the zuranolone 30 mg group compared with the placebo group.

(B) Forest plot of subgroup analysis of change from baseline in ISI subscale score

A mixed-effects model for repeated measures with unstructured covariance structure was applied to estimate study intervention difference between zuranolone and placebo; change from baseline was included as a response variable; study intervention, study identifier, time point, and interaction between study intervention and time point were included as fixed effects, with score at baseline, presence or absence of prior drug for depressive episodes and sex as covariates. *P < 0.05 (Nominal P-values are presented without adjustment for multiple comparisons and should be interpreted descriptively and as hypothesis-generating). Abbreviations: CI, confidence interval; SE, standard error; ISI, Insomnia Severity Index

# 3 Supplementary tables

**Supplementary Table 1.** Type III Tests of Fixed Effects for the MMRM Analysis

| Fixed Effect or Covariate | Num DF | Den DF | F-statistic | p-value |
| --- | --- | --- | --- | --- |
| Study intervention | 1 | 542 | 6.84 | 0.0092 |
| Time point | 8 | 504 | 73.08 | <.0001 |
| Interaction between study intervention and time point | 8 | 504 | 2.24 | 0.0233 |
| Study identifier | 1 | 552 | 0.86 | 0.3536 |
| HAM-D17 total score at Baseline | 1 | 553 | 14.83 | 0.0001 |
| Presence or absence of prior drug for depressive episodes | 1 | 552 | 0.18 | 0.6711 |
| Sex | 1 | 552 | 0.16 | 0.6882 |

Note: F-statistics and p-values are from Type III tests of fixed effects obtained from a MMRM. Num DF = Numerator degrees of freedom; Den DF = Denominator degrees of freedom (Kenward-Roger adjusted).
